# Multifaceted modulation of human opioid receptors by kratom alkaloids: binding affinity, functional selectivity, and allosteric activity

**DOI:** 10.3389/fphar.2026.1763551

**Published:** 2026-03-17

**Authors:** S. E. Hemby, M. Rangel-Grimaldo, S. McIntosh, J. Zheng, L. Flores-Bocanegra, T. N. Graf, R. A. Coover, N. H. Oberlies

**Affiliations:** 1 Department of Basic Pharmaceutical Sciences Fred Wilson School of Pharmacy High Point University, High Point, NC, United States; 2 Department of Chemistry and Biochemistry University of North Carolina at Greensboro, Greensboro, NC, United States

**Keywords:** 7-hydroxymitragynine, functional selectivity, kratom, Mitragyna speciosa, mitragynine, opioid receptor signaling, positive allosteric modulation, structure activity relationship

## Abstract

**Introduction:**

Kratom (Mitragyna speciosa) contains over 50 alkaloids, yet the pharmacological activity of most remains poorly defined, limiting our understanding of its therapeutic potential and safety profile.

**Methods:**

We conducted a comprehensive evaluation of both indole and oxindole alkaloids at human mu-, kappa-, and delta-opioid receptors (hMOR, hKOR, hDOR), integrating radioligand binding, cAMP inhibition, β-arrestin2 recruitment, [35S]GTPƔS assays, and molecular docking.

**Results:**

While the activity of major alkaloids like mitragynine and 7-hydroxymitragynine is well documented, we report detailed functional and structural characterization of lesser-known kratom alkaloids, including epiallo-isopaynantheine, isopaynantheine, mitraciliatine, and isospeciofoline. These compounds exhibited diverse receptor selectivity and functional profiles, ranging from G protein-biased agonism to mixed MOR antagonism/KOR agonism. Notably, speciophylline demonstrated positive allosteric modulation at hMOR without direct orthosteric binding – a mechanism not previously demonstrated experimentally for kratom alkaloids at human opioid receptors. Several oxindole alkaloids showed potent hMOR agonism with minimal β-arrestin2 recruitment, representing an extreme G-protein signaling bias that distinguishes them from classical opioids. Structure-activity analysis identified conserved pharmacophoric elements at C15 and C20 that govern receptor affinity and functional profile across both indole and oxindole scaffolds.

**Discussion:**

This systematic characterization at human opioid receptors reveals a pharmacologically diverse and structurally tunable class of natural products with potential as templates for developing opioid analgesics with improved therapeutic profiles.

## Introduction

Kratom [*Mitragyna speciosa* (Korth.) Havil. (Rubiaceae)] is a tropical tree native to Southeast Asia—particularly southern Thailand, Indonesia, and northern Malaysia—where it has long been cultivated for its medicinally active leaves (Brown et al., 2017). Traditionally, manual laborers consumed kratom to combat fatigue and enhance endurance during long hours of physical work. At higher doses, however, its use produces opioid-like effects, including analgesia, antitussive, antidiarrheal, and anti-inflammatory actions (Trakulsrichai et al., 2015; Mun et al., 2025). Kratom extracts have also served as opium substitutes, particularly during times of restricted opium availability (Jansen and Prast, 1988; Ward et al., 2011), and were used to alleviate opioid withdrawal symptoms (Boyer et al., 2007; Wilson et al., 2020). In recent years, kratom use has expanded globally, particularly in the United States, where it is consumed both recreationally and as a self-administered strategy to manage opioid dependence (Ward et al., 2011; Smith and Lawson, 2017; Grundmann et al., 2021; Smith et al., 2021). However, accumulating evidence of adverse effects, dependence liability, and rare fatalities, particularly involving adulterated products or polydrug use, underscores the need to define kratom’s molecular mechanisms of action (Swogger and Walsh, 2018; Corkery et al., 2019; Prozialeck et al., 2019).

Complex natural products such as kratom comprise a diverse array of bioactive constituents whose combined physiological effects can only be understood by the meticulous identification and characterization of individual compounds. Phytochemical studies have identified more than 50 indole and oxindole alkaloids in *M. speciosa* (Flores-Bocanegra et al., 2020; Todd et al., 2020), which together contribute to its pharmacological complexity. Alkaloid composition varies widely between kratom strains and regions of cultivation (Leon et al., 2009; Manwill et al., 2022; Sharma et al., 2025), likely accounting for differences in subjective effects and reinforcing the need for integrated pharmacological and botanical investigations to clarify the plant’s multifaceted bioactivity (Grundmann et al., 2024; Henningfield et al., 2024). Among these alkaloids, a subset has been pharmacologically characterized, revealing diverse and sometimes conflicting receptor interactions.

The pharmacological activity of *M. speciosa* arises primarily from its diverse alkaloid profile, which includes indole and oxindole compounds that act at multiple receptor systems (Kruegel et al., 2016; Obeng et al., 2020; Chear et al., 2021). The principal indole alkaloid, mitragynine (MG), and its oxidized metabolite 7-hydroxymitragynine (7-HMG), function as partial agonists at the μ-opioid receptor (MOR) and interact to varying degrees with δ- (DOR) and κ-opioid receptors (KOR) (Kruegel et al., 2016; Varadi et al., 2016). In preclinical studies, 7-HMG demonstrates substantially higher MOR affinity and analgesic potency than MG, with potency approximately 10-fold greater than morphine (Matsumoto et al., 2004; Kruegel and Grundmann, 2018). MG shows weaker MOR affinity but is metabolized to 7-HMG *in vivo* by human CYP3A4, indirectly enhancing opioid receptor activation (Kruegel et al., 2019). Notably, both MG and 7-HMG display G protein–biased agonism at MOR, preferentially activating G-protein pathways over β-arrestin recruitment, a signaling profile that has been hypothesized to underlie improved therapeutic outcomes (Kruegel et al., 2016; Varadi et al., 2016). However, the relationship between signaling bias and therapeutic outcomes is now recognized as more complex than initially proposed. For example, β-arrestin signaling alone does not account for all opioid-associated adverse effects, including respiratory depression. G-protein-mediated signaling can also contribute to both therapeutic and adverse outcomes, and differences in intrinsic efficacy and receptor reserve can give rise to apparent pathway bias without true pathway-selective engagement. Thus, G-protein bias should not be interpreted as inherently beneficial in the absence of quantitative bias analysis and *in vivo* validation.

Although several additional alkaloids, including speciociliatine, paynantheine, and speciogynine, have been investigated, their pharmacological profiles remain incompletely characterized (Kruegel and Grundmann, 2018; McCurdy et al., 2024). Some may modulate adrenergic and serotonergic signaling, though these actions are considered secondary to their opioid receptor activity (Kruegel et al., 2016; Obeng et al., 2020; Leon et al., 2021). Importantly, speciociliatine has been described both as a full agonist at human MOR (hMOR) comparable to morphine (Chear et al., 2021) and as a weak antagonist at hMOR and mouse MOR (mMOR), respectively (Kruegel et al., 2016; Zhou et al., 2021). These discrepancies emphasize the need for detailed, standardized characterization across consistent receptor models. The oxindole alkaloids of kratom have received considerably less attention than their indole counterparts, despite comprising a substantial portion of the plant’s alkaloid content; systematic evaluation of their binding affinities and functional profiles at human opioid receptors is lacking. Moreover, all prior pharmacological studies of kratom alkaloids have focused exclusively on orthosteric receptor interactions. Whether any of these compounds can modulate opioid receptor function through allosteric mechanisms, an increasingly important concept in opioid pharmacology, has not been examined.

In addition to these gaps in alkaloid characterization, kratom alkaloids display species-specific pharmacology that complicates the interpretation of preclinical findings and their translation to human therapeutic applications. For example, MG acts as a partial agonist at hMOR but as a competitive antagonist at mMOR, while 7-HMG behaves as a partial agonist at hMOR yet functions more like a full agonist at mMOR (Kruegel et al., 2016; Varadi et al., 2016). These differences likely reflect interspecies variation in receptor conformation, G-protein coupling efficiency, and receptor reserve. Species differences extend to metabolic pathways as well. For example, while MG is efficiently converted to 7-HMG by human CYP3A4, this bioactivation is less efficient in rodents (Kruegel et al., 2019; Kamble et al., 2020). Additionally, MG’s signaling profile at hMOR, characterized by minimal β-arrestin-2 recruitment, may not be conserved across species (Ellis et al., 2020). Together, these pharmacokinetic and pharmacodynamic differences underscore the challenges of extrapolating rodent data to humans and highlight the critical importance of evaluating kratom alkaloids using human opioid receptor models.

Despite the growing body of research on kratom’s major alkaloids, a systematic evaluation of both indole and oxindole alkaloids at human opioid receptor subtypes remains lacking. The current study comprehensively evaluates kratom alkaloids—including several indole and oxindole compounds not previously characterized at human opioid receptors—for their binding affinities and functional activities at hMOR, hKOR, and hDOR. By integrating radioligand binding assays, functional assays (cAMP inhibition, β-arrestin-2 recruitment, [^35^S]GTPγS), and molecular docking, we aim to clarify their receptor selectivity, signaling bias, and structure–activity relationships, while also probing for potential allosteric activity. This systematic characterization will provide a foundation for understanding both the therapeutic potential and the abuse liability of *M. speciosa* alkaloids, informing future clinical research and regulatory decisions.

## Materials and Methods

### Compounds

*Kratom Alkaloids. I*ndole and oxindole alkaloids were obtained through isolation, semi-synthesis, or commercial purchase. Mitragynine (MG), speciociliatine, speciociliatine-*N* (4)-oxide, mitraciliatine, speciogynine, paynantheine, isopaynantheine, epiallo-isopaynantheine, epiallo-isopaynantheine-*N* (4)-oxide, isopaynantheine-*N* (4)-oxide, mitragynine-*N* (4)-oxide, corynoxine A, corynoxine B, isospeciofoleine, isorotundifoleine, speciofoline, three-epicorynoxine B, three-epirhynchophylline, and corynoxeine were isolated from *M. speciosa* leaf material and fully characterized by NMR and MS, with spectral data matching literature values (Flores-Bocanegra et al., 2020). 7-hydroxymitragynine (7-HMG) was prepared via semi-synthesis from MG using established procedures (Kruegel et al., 2019) and characterized as previously described (Flores-Bocanegra et al., 2020). Purity was independently confirmed in-house by HPLC prior to use. Rhynchophylline was purchased from Carbosynth Ltd. (Compton, Berkshire, UK); mitraphylline from AOBIOUS (Gloucester, MA), speciophylline from ChromaDex/Niagen Bioscience (Rockville, MD), isomitraphylline from BOC Sciences (New York, NY), and ajmalcine from AdipoGen Life Sciences (San Diego, CA). Additional quantities of corynoxine A and speciophylline were also purchased from Target Molecule Corporation (Wellesley Hills, MA) and Alfa Chemistry (Ronkonkoma, NY), respectively, to ensure sufficient yield for testing. Certificates of analysis confirming identity and purity (>90%) were provided by the respective vendors. Vendor COAs are available upon request.

*Reference Compounds.* Reference opioid agonists and antagonists were obtained from commercial sources. [D-Ala^2^, *N*-MePhe^4^, Gly-ol]-enkephalin (DAMGO), [D-Pen^2^, D-Pen^5^]-enkephalin (DPDPE), naloxone hydrochloride, and nor-binaltorphimine dihydrochloride were purchased from Sigma-Aldrich (St. Louis, MO). Fentanyl citrate, buprenorphine hydrochloride, morphine sulfate, methadone hydrochloride, hydrocodone bitartrate, and oxycodone hydrochloride were obtained from Fagron (St. Paul, MN). Endomorphin I was purchased from Tocris Bioscience (Minneapolis, MN), and U50,488 from R&D Systems (Minneapolis, MN).

*Radioligands.* [^3^H]-DAMGO (specific activity: 50 Ci/mmol), [^3^H]-Deltorphin II (specific activity: 45 Ci/mmol), and [^3^H]-U-69,593 (specific activity: 40 Ci/mmol) were purchased from PerkinElmer (Waltham, MA) and used for radioligand binding assays at hMOR, hDOR, and hKOR, respectively.

### Competitive radioligand binding assays

CHO-K1 cells expressing hMOR, hDOR and hKOR (Perkin Elmer ValiScreen®: ES-542-C, RBHODM-K, ES-541-C) were grown in F-12 Hams with 5% FBS, and penicillin/streptomycin (with supplemented G418 every three to four passages for selection). Cells were washed in ice-cold PBS and scraped from the cell culture plate in a cold 50 mM Tris-HCl buffer, pH 7.4. The cells were pelleted at 5,200 × g for 10 min at 4 °C. The supernatant was discarded, and the pellet was washed with additional Tris-HCl buffer, sonicated, and centrifuged at 24,000 × g for 40 min at 4 °C. The pellet was re-suspended in Tris-HCl buffer, sonicated, and aliquoted for storage at −80 °C until use. A Pierce™ BCA Protein Assay was utilized to determine protein concentration in the membrane, according to the manufacturer’s instructions. Membranes were evaluated in saturation experiments to determine the optimal concentration for radioligand binding and functional assays.

A competitive binding assay was used to determine the Ki of each compound, as in previous reports (Giacometti et al., 2013). Twenty-5 μg of cell membrane were diluted in 50 mM Tris-HCl, containing 0.1% BSA, pH 7.4, and pipetted into a 96-well plate with total reaction volumes of approximately 200 μL per well. Test compounds were reconstituted in DMSO and added to the reaction plate. Radioligands specific to each receptor were then added at the following final concentrations: [^3^H]-DAMGO at 1.0 nM (hMOR), [^3^H]-U-69,593 at 1.0 nM (hKOR), and [^3^H]-Deltorphin II at 2.0 nM (hDOR). These concentrations approximated or were below the respective Kd values (2.5, 1.5, and 9.0 nM) to minimize radioligand depletion. The reaction was incubated for 90 min at 37 °C before transfer onto GF/B filter plates (Perkin Elmer). The plates were washed 10x with cold buffer and dried for 15 min at 50 °C. MicroScint20 was then added to each well, the plates were sealed, and the counts per minute were quantified on the TopCount NXT Microplate Scintillation counter (Perkin Elmer). The percent displacement of the radiolabeled compound was calculated with the following equation:

$$\%\;Displacement=100-\frac{\left(\text{cpm}\;\text{of}\;\text{test}\;\text{compound}-\text{cpm}\;\text{of}\;\text{non}‐\text{specific}\right)}{\left(\text{cpm}\;\text{of}\;\text{total}\;\text{bound}-\text{cpm}\;\text{of}\;\text{non}‐\text{specific}\right)}$$

Unlabeled DAMGO, U-69,593, and DPDPE were used to determine non-specific control counts, while vehicle (DMSO) was added to determine total radioligand binding on each plate. A series of three-fold dilutions were used for each test compound starting at 10 μM. A control curve of naloxone was included on each plate as an internal control. Displacement curves were generated and Ki values were calculated as described in *Data Analysis and Statistics*.

### Functional activity assays

#### cAMP accumulation

The assay served as a primary functional screen for receptor activation due to its high signal-to-noise ratio resulting from intracellular signal amplification, thus enabling the detection of weak agonist activity. Inhibition of forskolin-stimulated cAMP was determined using DiscoverX Hit Hunter assays in CHO-K1 cells stably expressing hMOR, hDOR, or hKOR either in-house or at Eurofins DiscoverX. The same assay protocol was used at both sites. The assay is based on enzyme-fragment-complementation (EFC) in which β-galactosidase is split into an Enzyme Acceptor (EA) and an Enzyme Donor (ED) fragment. The ED fragment is conjugated to cAMP and competes with endogenous cAMP for binding to a cAMP-specific antibody. Unbound ED-cAMP complements with EA to form active β-galactosidase, which hydrolyzes a chemiluminescent substrate to generate a luminescent signal proportional to cellular cAMP levels. Luminescence was measured using a SpectraMax iD5 or SpectraMax M5e microplate reader (Molecular Devices). Cells were plated at approximately 3 × 10^4^ cells per well in a 96-well plate (Eurofins DiscoverX) or 1 × 10^4^ in a 384-well plate (in-house) and cultured for 15–20 h at 37 °C and 5% CO_2_ prior to compound treatment. Reaction volumes were format-dependent. Assays were performed according to the manufacturer’s protocol.

For assays run in agonist mode, cells were treated with the indicated compounds at various concentrations (10^–5^ to 10^–12^ *M*) in the presence of 15 μM forskolin for 30 min at 37 °C and the inhibition of cAMP accumulation was assessed. To normalize assay responses, DAMGO, DADLE, and dynorphin A served as reference agonists for hMOR, hKOR and hDOR, respectively. In antagonist mode, cells were treated with test compounds over a concentration range of 10^–12^ to 10^–5^ *M* in the presence of 15 µM forskolin for 15 min at 37 °C, followed by the addition of the EC_80_ concentration of the receptor agonist and incubation for 30 min at 37 °C. Receptor agonists for hMOR, hKOR and hDOR were met-enkephalin, dynorphin A and DADLE, respectively. To normalize assay responses, reference antagonists for hMOR, hKOR, and hDOR were naloxone, nor-binaltorphimine, and naltriben, respectively. To assess speciophylline in positive allosteric mode, cells were treated with test compounds over a concentration range of 10^–12^ to 10^–5^ *M* in the presence of 15 μM forskolin for 15 min at 37 °C, followed by the addition of the EC_20_ concentration of met-enkephalin and incubation for 30 min at 37 °C. EC_50_, IC_50_, and E_max_ values were determined as described in *Data Analysis and Statistics*.

#### β-arrestin-2 recruitment

Compounds that exhibited positive inhibition of forskolin cAMP accumulation were further assessed for their ability to inhibit forskolin-stimulated β-arrestin2 recruitment using DiscoverX PathHunter assays in CHO-K1 cells stably expressing hMOR and hDOR, or in U2OS cell stably expressing hKOR either in-house or at Eurofins DiscoverX. Similar assay protocols were used at both sites. The assay provides a complementary measure of GPCR activation via arrestin-mediated (non-canonical) signaling, helping assess ligand-biased activity alongside G-protein pathways. Luminescence was measured using a SpectraMax iD5 or M5e microplate reader (Molecular Devices). Cells were seeded at approximately 2.5 × 10^4^ cells per well in a 96-well plate or 5 × 10^3^ cells per well 384-well plate (in-house) and cultured for 48 h at 37 °C and 5% CO_2_ prior to compound treatment. Reaction volumes were format-dependent. Assays were performed according to the manufacturer’s protocol.

For assays run in agonist mode, cells were treated with the indicated compounds at concentrations ranging from 10^–5^ to 5 × 10^−10^ M in the presence of 15 μ*M* forskolin for 90 min at 37 °C. In antagonist mode, cells were treated with test compounds at concentrations ranging from 10^–5^ to 5 × 10^−10^ M for 30 min at 37 °C, followed by the addition of the EC_80_ concentration of the receptor agonist and an additional incubation for 90 min at 37 °C. Receptor agonists for hMOR, hKOR and hDOR were met-enkephalin, dynorphin A and DADLE, respectively. Met-enkephalin, DADLE, and dynorphin A served as reference agonists for hMOR, hDOR, and hKOR, respectively. To assess speciophylline in positive allosteric mode, cells were treated with test compounds over a concentration range of 10^–4^ to 5 × 10^−9^ M in the presence of 15 µM forskolin for 15 min at 37 °C, followed by the addition of the EC_20_ concentration of the met-enkephalin and incubation for 90 min at 37 °C. EC_50_, IC_50_, and E_max_ values were determined as described in *Data Analysis and Statistics*.

#### [^35^S] GTPγS binding

Based on their mixed agonist-antagonist profiles observed in cAMP assays, mitraciliatine and isopaynantheine were selected for further evaluation by [^35^S]GTPγS binding. Eurofins DiscoverX performed functional [^35^S]GTPγS binding assays to evaluate the agonist and antagonist activity of mitraciliatine and isopaynantheine at hMOR and hKOR using their validated radioligand platform. Assays were conducted in CHO-K1 cells stably expressing hMOR and in Chem-1 cells stably expressing hKOR. In agonist mode, membrane preparations from cells were incubated with increasing concentrations of test compounds (10^–8^ to 10^–5^ M) in incubation buffer (20 mM HEPES, pH 7.4, 150 mM NaCl, 10 mM MgCl_2_, 1 mM DTT, 1 mM EDTA, 10 μg/mL saponin) containing GDP (30 µM) and [^35^S]GTPγS (0.05 nM) at 30 °C for 30 min. Post-filtration wash steps for [^35^S]GTPγS binding assays followed Eurofins DiscoverX validated protocols. Filters were washed with ice-cold buffer to remove unbound radioactivity; specific wash volumes and number of wash cycles are proprietary and available from the vendor upon request. Responses were normalized to basal activity (vehicle control) and expressed as a percentage of the response produced by a reference full agonist (DAMGO for hMOR and U69593 for hKOR). In antagonist mode, test compounds were co-incubated with an EC_80_ concentration of the aforementioned reference agonists to assess their ability to inhibit agonist-induced G-protein activation. To normalize assay responses, reference antagonists for hMOR, hKOR, and hDOR were naltrexone, nor-binaltorphimine, and naltrindole, respectively. The assay conditions were otherwise identical to those described above. EC_50_, IC_50_, and E_max_ values were determined as described in *Data Analysis and Statistics*.

#### *In silico* molecular docking

Protein Preparation. The cryo-EM structure of human MOR in complex with mitragynine pseudoindoxyl (PDB: 7T2G) (Qu et al., 2023) was used for docking of agonists and partial agonists. The inactive-state crystal structure of mouse MOR (PDB: 4DKL) (Manglik et al., 2012) was used for docking of antagonists. Protein structures were prepared in AutoDockTools 1.5.7 by removing co-crystallized ligands, water molecules, and non-essential ions. Polar hydrogens were added, and Kollman charges were assigned to all atoms at pH 7.4.

Ligand Preparation. Three-dimensional structures of kratom alkaloids were generated from SMILES strings using ChemDraw and energy-minimized using the Chem3D MM2 force fields. For alkaloids containing a basic tertiary amine, the protonated form was used to reflect physiological pH. Rotatable bonds were assigned automatically, and Gasteiger charges were calculated in AutoDockTools.

Docking Protocol. Molecular docking was performed using AutoDock 4.2.6 (Morris et al., 2009) with the Lamarckian genetic algorithm (LGA). For the active-state MOR structure (7T2G), the grid box was centered at 75.272, 105.533, 58.222 (x, y, z) with dimensions of 46 × 48 × 58 points and 0.4 Å spacing, encompassing the orthosteric binding pocket. For the inactive-state structure (4DKL), the grid box was centered at −26.361, −10.763, −12.044 (x, y, z) with dimensions of 56 × 58 × 62 points and 0.375 Å spacing. Docking parameters included 50 independent genetic algorithm runs per compound, a population size of 300, and a maximum of 5 × 10^6^ energy evaluations per run.

Validation. The docking protocol was validated by re-docking the co-crystallized ligand (mitragynine pseudoindoxyl) into the 7T2G binding site. The top-ranked pose reproduced the crystallographic binding mode with an RMSD of 0.00 Å, confirming the reliability of the docking parameters.

Pose Selection and Analysis. Docked poses were clustered using an RMSD tolerance of 2.0 Å. The lowest-energy pose from the most populated cluster was selected as the representative binding mode for each compound. Protein–ligand interactions, including hydrogen bonds (distance cutoff ≤3.5 Å, angle ≥120°), hydrophobic contacts, and π-stacking interactions, were analyzed and visualized using PyMOL 2.5 (Schrödinger, 2023). Hydrogen bond distances reported in the Results represent heavy atom distances (e.g., N–O or O–O).

### Data analysis and statistics

All concentration–response data were analyzed using GraphPad Prism (version 10.6.1, GraphPad Software, San Diego, CA). Displacement and concentration–response curves were fitted using a four-parameter logistic model with the following equation:

$$Y=Bottom+\frac{\left(Top-Bottom\right)}{1+10^{\left(LogEC_{50}-X\right)\times HillSlope}}$$

For radioligand binding assays, Ki values were calculated from IC_50_ values using the Cheng-Prusoff equation:

$$K_{i}=\frac{IC_{50}}{1+\frac{\left[L\right]}{K_{d}}}$$

where [L] is the concentration of radioligand and Kd is the equilibrium dissociation constant determined from saturation binding experiments. The Kd values for [^3^H]-DAMGO, [^3^H]-U-69,593, and [^3^H]-Deltorphin II at hMOR, hKOR, and hDOR were 2.0 nM, 1.5 nM, and 9.0 nM, respectively (determined in-house; data not shown).

For functional assays (cAMP accumulation, β-arrestin-2 recruitment, and [^35^S]GTPγS binding), responses were normalized to reference compounds to enable cross-assay comparisons. In agonist mode, responses were expressed as a percentage of the maximal effect (E_max_) produced by the reference full agonist. In antagonist mode, responses were expressed as a percentage of the maximal inhibition produced by the reference antagonist. EC_50_ (agonist potency), IC_50_ (antagonist potency), and Emax values were derived from the fitted curves. cAMP accumulation assays were performed in a minimum of two independent experiments, each conducted with two to four technical replicates per concentration. β-arrestin-2 recruitment assays were performed in at least two independent experiments, each with two to three technical replicates per concentration. [^35^S]GTPγS binding assays were performed with triplicate determinations at each concentration. Data are presented as mean ± SEM. Compounds were considered inactive if EC_50_ or IC_50_ values exceeded 10 µM or if Emax was <10% of the reference compound.

Commercially validated cell lines from the assay manufacturer were used for each receptor-assay pair. Specifically, CHO-K1 cells were used for hMOR and hDOR in β-arrestin-2 recruitment assays, while U2OS cells were used for hKOR. For [^35^S]GTPγS binding assays, CHO-K1 cells were used for hMOR and Chem-1 cells for hKOR. Variations in cellular background, receptor expression levels, and receptor reserve may affect absolute potency and efficacy values. Therefore, cross-receptor comparisons should be made with this consideration in mind.

Positive allosteric modulation at hMOR was operationally defined using established submaximal agonist and binding-based criteria (Burford et al., 2013; Burford et al., 2015; Livingston et al., 2018). Specifically, compounds were classified as positive allosteric modulators if they (i) lacked intrinsic agonist activity when applied alone, (ii) potentiated the response to a submaximal (EC_20_) concentration of an orthosteric agonist in the cAMP assay, and/or (iii) enhanced orthosteric agonist binding in radioligand assays. This experimental strategy is widely used for the identification of μ-opioid receptor positive allosteric modulators, particularly for low-affinity or pathway-selective modulators, and does not require demonstration of agonist concentration–response curve shifts.

## Results

### Receptor binding

The binding affinities of selected indole alkaloids from kratom were assessed at hMOR, hKOR, and hDOR using competitive radioligand binding assays. Results are summarized in Table 1. For classification purposes, affinities were categorized as follows: high (Ki < 50 nM), moderate (Ki: 50–500 nM), weak (Ki: 500–5000 nM), or negligible (Ki > 5000 nM or not detectable).

**TABLE 1:** Binding affinity (K_i_) of kratom alkaloids and opioids at human opioid receptors.

Compound	Affinity (K_i_ ± SEM, nM)		
	hMOR	hKOR	hDOR
Indole			
7-hydroxymitragynine	15.1 ± 3.7	113 ± 37	137.3 ± 21.3
Speciociliatine	49 ± 13	312 ± 56	--
Epiallo-isopaynantheine	155 ± 15	120 ± 8	--
Mitraciliatine	226 ± 26	108 ± 16	--
Mitragynine	238 ± 28	482 ± 29	--
Isopaynantheine	262 ± 36	130 ± 12	2876 ± 848
Speciogynine	472 ± 65	--	3355 ± 102
Payantheine	1270 ± 141	--	9029 ± 2436
Mitragynine-*N*(4)-oxide	--	145 ± 12	​
Isopaynantheine-*N*(4)-oxide	--	549 ± 74	--
Speciociliatine-*N*(4)-oxide	--	--	6000 ± 965
Ajmalicine	--	--	--
Oxindole			
Corynoxine A	5.4 ± 0.3	2310 ± 325	--
Isospeciofoline	167 ± 13.3	--	8050 ± 1340
Corynoxine B	118.3 ± 12.1	--	--
3-epicorynoxine B	5050 ± 1403	--	6665 ± 3022
3-epirhyncophylline	6226 ± 771	--	--
Corynoxeine	7187 ± 865	--	--
Rhynchophylline	--	--	4248 ± 551
Speciofoline	--	--	7987 ± 992
Isomitraphylline	--	--	--
Isorotundifoline	--	--	--
Mitraphylline	--	--	--
Speciophylline (Uncarine D)	--	--	--
Controls			
Naloxone	1.6 ± 0.2	6.6 ± 0.3	75 ± 9
Morphine	1.5 ± 0.04	140 ± 16	1830 ± 495
Fentanyl	0.8 ± 0.1	406 ± 39	718 ± 127
Methadone	1.9 ± 0.1	402 ± 27	1285 ± 500
Norbinaltorphimine	​	0.9 ± 0.1	​
DPDPE	​	​	62 ± 5

Competitive binding assays were conducted with the indicated compounds against [^3^H]-DAMGO (MOR), [^3^H]-U69,593 (KOR), and [^3^H]-DPDPE (DOR) in membranes of CHO-K1, cells stably expressing the respective cloned human opioid receptor. Results are presented as nM (mean ± SEM). – indicates >10 μM. blank cell indicates not tested.

Among the indole alkaloids tested, 7-HMG exhibited high affinity for hMOR (Ki = 15.1 ± 3.7 nM), and was also the only compound with appreciable binding at all three opioid receptor subtypes, displaying moderate affinity at hDOR (Ki = 137.3 ± 21.3 nM) and weak affinity at hKOR. Several alkaloids displayed dual hMOR/hKOR affinity and negligible binding at hDOR. Speciociliatine also displayed high affinity at hMOR (Ki = 49 ± 13 nM) with moderate binding at hKOR. MG and speciogynine showed moderate and weak hMOR affinity, respectively. Isopaynantheine, epiallo-isopaynantheine, and mitraciliatine exhibited moderate affinity at both hMOR and hKOR, while paynantheine showed moderate hMOR and weak hKOR binding. None of these compounds displayed appreciable hDOR affinity. The *N* (4)-oxide derivatives exhibited altered selectivity profiles. Isopaynantheine-*N*(4)-oxide and mitragynine-*N*(4)-oxide lacked measurable hMOR binding but retained moderate hKOR affinity, suggesting receptor selectivity influenced by nitrogen oxidation. In contrast, speciociliatine-*N*(4)-oxide showed very weak affinity for hDOR only.

Among the oxindole alkaloids tested, corynoxine A exhibited the highest affinity of any alkaloid tested at hMOR (Ki = 5.4 ± 0.3 nM), with weak binding at hKOR (2310 ± 325 nM) and hDOR (2932 ± 1110 nM). Corynoxine B and isospeciofoline exhibited moderate hMOR binding affinities with negligible binding at other subtypes. Corynoxeine, three-epirhynchophylline, and three-epicorynoxine displayed weak hMOR binding only. Several oxindole alkaloids, including speciofoline, rhynchophylline, isomitraphylline, isorotundifoline, ajmalicine, and mitraphylline, did not exhibit measurable binding at any receptor. Speciophylline (also known as Uncarine D) lacked detectable orthosteric binding at any of the receptors, but enhanced [^3^H]DAMGO binding at hMOR in a concentration-dependent manner, consistent with positive allosteric modulation.

### Functional assays

Compounds exhibiting measurable binding affinity (Ki < 2 µM) were evaluated for functional activity using cAMP accumulation and β-arrestin-2 recruitment assays (Table 2). For classification, potency was categorized as high (EC_50_/IC_50_ < 50 nM), moderate (51–500 nM), weak (501–5000 nM), or inactive (>5001 nM). Efficacy was classified as full (Emax ≥85%), partial (Emax 15%–84%), or minimal (<15%) relative to reference agonists.

**TABLE 2:** Inhibition of forskolin-stimulated cAMP Accumulation in CHO1 cells expressing human opioid receptors.

Compound	hMOR		hKOR		hDOR	
	EC50, nM (*Emax,%)*	IC50, nM (*Emax,%)*	EC50, nM (*Emax,%)*	IC50, nM (*Emax,%)*	EC50, nM (*Emax,%)*	IC50, nM (*Emax,%)*
Indole						
7-hydroxymitragynine	9.7 ± 0.8 (*97.6 ± 0.6)*	​	372.4 ± 90.2 (*63.7 ± 11.5*)	--	93.6 ± 23.1 (*89.2 ± 2.6*)	​
Speciociliatine	127.1 ± 24 (*69.8 ± 3.8)*	--	715.1 ± 200 (93*.6 ± 1.3)*	--	​	​
Epiallo-isopaynantheine	--	257.2 ± 100.7 (*44.9 ± 3.9)*	​	​	​	​
Mitraciliatine	--	1890 ± 256 (*89.9 ± 3.5)*	142.8 ± 10.2 (*101.6 ± 0.8)*	​	​	​
Mitragynine	396.0 ± 22.6 (*69.4 ± 1.4)*	​	974 ± 177 (*83.4 ± 1.7*)	--	​	​
Isopaynantheine	--	1778 ± 236 (*87.3 ± 3.6)*	139.6 ± 8.5 (*101.9 ± 1.0)*	​	​	​
Speciogynine	2231 ± 839 (*31.8 ± 1.6)*	--	--	--	​	​
Paynantheine	653.2 ± 73.8 (*29.2 ± 3.2)*	--	--	--	​	​
Mitragynine-*N*(4)-oxide	​	​	120 ± 42.5 (*95.2 ± 0.3)*	--	​	​
Isopaynantheine-*N*(4)-oxide	​	​	>10,000	--	​	​
Oxindole						
Corynoxine A	34.3 ± 2.6 (*91.4 ± 3.0)*	--	​	​	​	​
Isospeciofoline	104 ± 7.3 (*77.8 ± 3.3)*	--	​	​	​	​
Corynoxine B	193.1 ± 15.2 (*80.9 ± 5.4)*	--	​	​	​	​
Speciophylline	^#^ PAM	--	​	​	​	​
Control						
[Met] enkephalin	2.5 ± 0.7 (*102.5 ± 0.7)*	​	​	​	​	​
DAMGO	2.2 ± 0.1 (*89.4 ± 1.4)*	​	​	​	​	​
Naloxone	​	9.4 ± 3.5 (*100.8 ± 0.2*)	​	​	​	​
Dynorphin A	​	​	0.3 ± 0.06 (*100.8 ± 0.2*)	​	​	​
Nor-BNI	​	​	​	22.4 ± 7.8 (*100.6 ± 4.7*)	​	​
DADLE	​	​	​	​	0.29 ± 0.03 (*99.0 ± 0.5*)	​
Naltriben mesylate	​	​	​	​	​	8.67 ± 0.30 (*101.5 ± 6.6*)

Agonist activity indicated by EC_50_ values, maximal efficacy (E_max_) relative to DAMGO, dynorphin A or DADLE, for MOR, KOR, and DOR, respectively. Antagonist activity indicated by IC_50_ values for the respective reference agonist at EC_80_. Blank cell indicates not tested. Data shown are mean ± SEM.

^#^No intrinsic agonist activity. Positive allosteric modulator that potentiated met-enkephalin (EC_20_ = 0.66 nM)–induced cAMP, inhibition with EC_50_ = 27.1 ± 4.5 µM. See Figure 3B.

#### Inhibition of forskolin-stimulated cAMP accumulation

**FIGURE 1:**
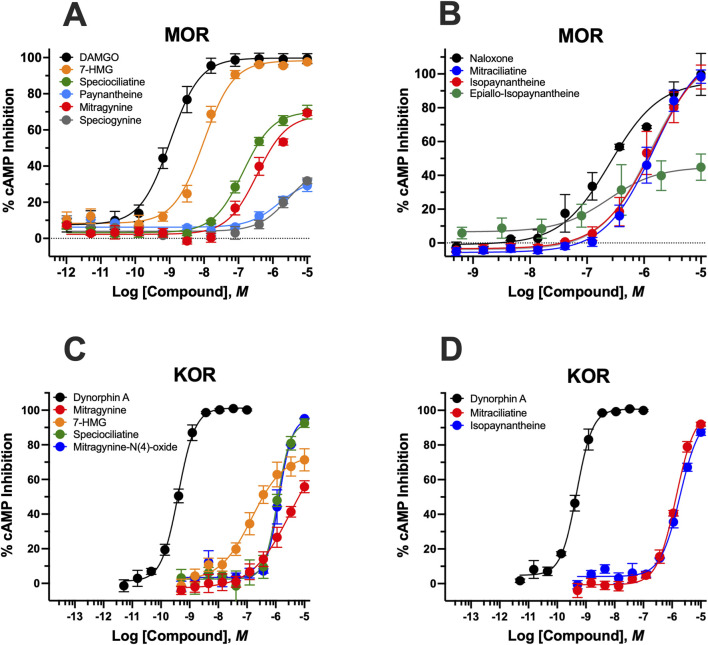
Inhibition of forskolin-induced cAMP accumulation by kratom indole alkaloids at hMOR and hKOR. **(A)** Concentration-response curves showing the effects of the MOR full agonist DAMGO and various indole kratom alkaloids on the inhibition of forskolin-stimulated cAMP accumulation in hMOR-expressing cells. Data are normalized to the maximal response produced by DAMGO and expressed as % of DAMGO E_max_. **(B)** Antagonist activity of naloxone and select indole kratom alkaloids evaluated by their ability to reverse the inhibitory effect of met-enkephalin (E_80_ concentration) on forskolin-stimulated cAMP accumulation. Responses are expressed as % of naloxone E_max_, representing the maximal reversal of DAMGO-mediated inhibition. **(C,D)** Concentration-response curves showing the effects of the KOR full agonist Dynorphin A and indole kratom alkaloids on inhibition of forskolin-stimulated cAMP accumulation. Data are expressed as % of Dynorphin E_max_. Data represent mean ± SEM.

*Indole alkaloids* (Figure 1; Table 2) 7-HMG exhibited the broadest functional activity, acting as the only kratom alkaloid with measurable efficacy at all three opioid receptor subtypes.At hMOR, 7-HMG displayed high potency as a full efficacy agonist (EC_50_ = 13.6 ± 0.9 nM, E_max_ = 85.9 ± 0.4%). It also showed moderate potency with full efficacy at hKOR (EC_50_ = 440.1 ± 7.0 nM, E_max_ = 104.8 ± 6.1%) and hDOR (EC_50_ = 150.8 ± 22.6 nM, E_max_ = 82.7 ± 5.6%). Speciociliatine functioned as a partial agonist at hMOR with moderate potency and at hKOR with weak potency. MG displayed a profile similar to speciociliatine, but with lower efficacy at hKOR. Paynantheine and speciogynine exhibited weak potency and minimal efficacy at hMOR (EC_50_ > 1 μM; E_max_ < 32%). Among the *N* (4)-oxide derivatives, mitragynine-*N* (4)-oxide showed moderate potency as a full efficacy hKOR agonist. Speciociliatine-*N* (4)-oxide lacked measurable activity at any opioid receptor.

Three indole alkaloid, mitraciliatine, isopaynantheine and epiallo-isopaynantheine, functioned as antagonists at the hMOR. Mitraciliatine and isopaynantheine exhibited weak potency and high efficacy antagonism, while epiallo-isopaynantheine showed moderate potency and partial antagonist activity. Notably, mitraciliatine and isopaynantheine also functioned as moderate potency, full efficacy agonists at hKOR, representing a mixed antagonist/agonist profile across receptor subtypes.

### Oxindole Alkaloids

**FIGURE 2:**
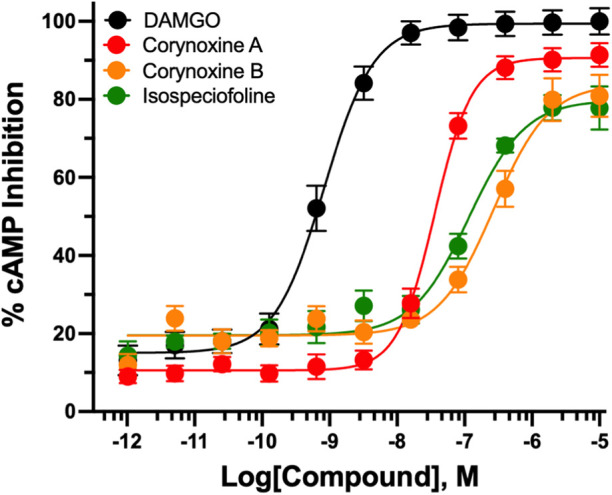
Inhibition of forskolin-induced cAMP accumulation by kratom oxindole alkaloids at hMOR*.* Concentration-response curves showing the effects of the MOR full agonist DAMGO and various oxindole kratom alkaloids on the inhibition of forskolin-stimulated cAMP accumulation in MOR-expressing cells. Data are normalized to the maximal response produced by DAMGO and expressed as % of DAMGO E_max_. Data represent mean ± SEM.

Table 2, Figure 2 oxindole alkaloids displayed a predominantly hMOR-selective functional profile, with a limited subset showing potent agonism and minimal activity at hKOR or hDOR. Corynoxine A exhibited the highest potency as a full efficacy agonist at hMOR (EC_50_ = 37.9 ± 3.1 nM, E_max_ = 88.3 ± 0.6%), followed by isospeciofoline (EC_50_ = 118.3 ± 12.1 nM, E_max_ = 85.6 ± 0.6%) and corynoxine B (EC_50_ = 276.1 ± 32.1 nM, E_max_ = 90.0 ± 0.4%).

**FIGURE 3:**
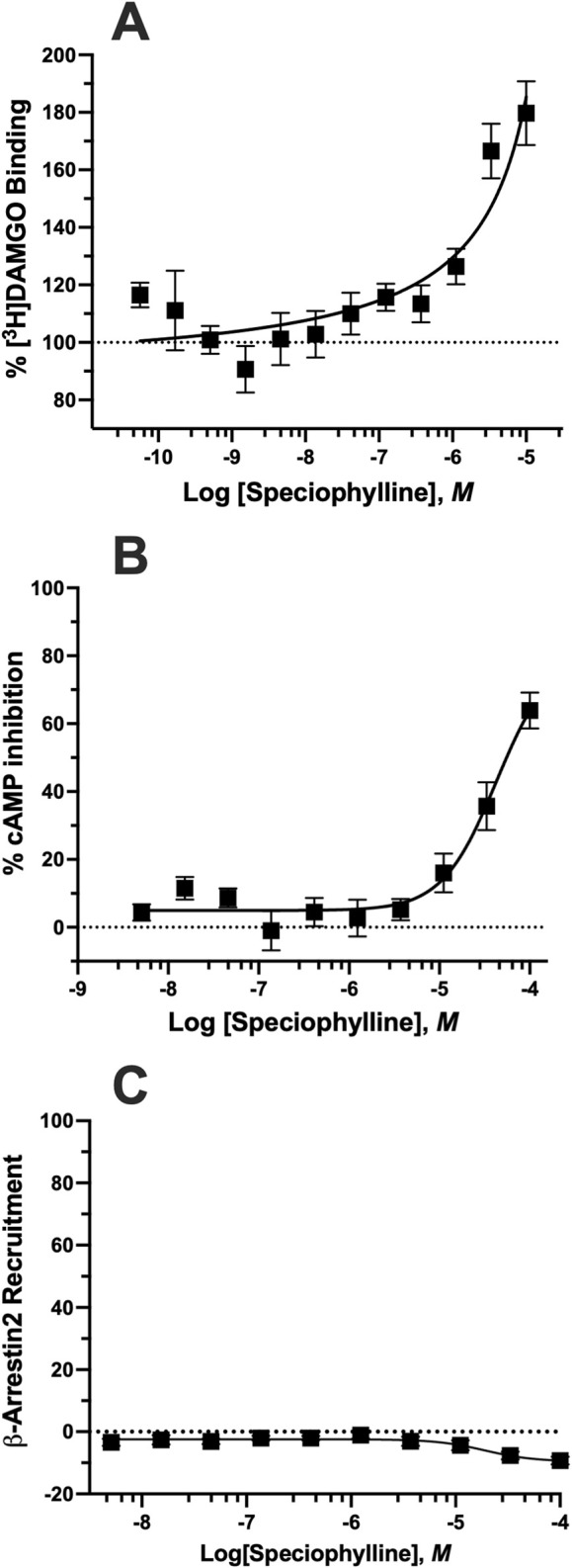
*Positive allosteric modulation by speciophylline at the hMOR*. **(A)** Competitive binding assay demonstrating that speciophylline enhances the binding of the orthosteric agonist [^3^H]DAMGO at the μ-opioid receptor, indicative of positive allosteric modulation. **(B)** Speciophylline enhanced met-enkephalin-induced inhibition of forskolin-stimulated cAMP accumulation in hMOR-expressing cells. Speciophylline was co-applied with E_20_ of met-enkephalin (i.e., the concentration producing ∼20% maximal inhibition of cAMP), allowing for sensitive detection of positive allosteric modulation. **(C)** Speciophylline failed to enhance recruitment of β-arrestin2. The potentiation of met-enkephalin’s effect in the presence of speciophylline supports its role as a G-protein biased-positive allosteric modulator of μ-opioid receptor signaling.

*Positive allosteric* modulation *by speciophylline*. Speciophylline was selected for further evaluation as a potential positive allosteric modulator because, unlike other oxindole alkaloids examined in this study, it lacked detectable orthosteric binding at hMOR yet uniquely enhanced orthosteric agonist binding (Speciophylline exhibited a unique pharmacological profile distinct from other kratom alkaloids (Table 2; Figure 3). It lacked intrinsic agonist activity at hMOR in cAMP assays; however, it functioned as a PAM, enhancing [^3^H]DAMGO binding in a concentration-dependent manner (Figure 3A) and potentiating met-enkephalin (EC_20_ = 0.66 nM) induced forskolin stimulated cAMP inhibition with an EC_50_ of 27.1 ± 4.5 µM (Figure 3B). Speciophylline did not exhibit detectable orthosteric binding or intrinsic functional activity at hKOR or hDOR under the conditions tested, and therefore, PAM analyses were focused on hMOR, where modulatory effects were observed. Notably, speciophylline failed to enhance β-arrestin-2 recruitment (Figure 3C), indicating selective modulation of G-protein signaling pathways. These results demonstrate that speciophylline acts as a G-protein-biased positive allosteric modulator at hMOR without direct orthosteric binding, a mechanism not previously demonstrated experimentally for kratom alkaloids at human opioid receptors.

Consistent with established criteria for μ-opioid receptor positive allosteric modulation, speciophylline lacked intrinsic agonist activity yet potentiated met-enkephalin–induced cAMP inhibition under submaximal (EC_20_) agonist conditions (Burford et al., 2013; Burford et al., 2015). In parallel, speciophylline enhanced [^3^H]DAMGO binding in a concentration-dependent manner, providing independent evidence of positive cooperativity at the receptor level (Livingston et al., 2018). Together, these functional and binding data satisfy widely accepted operational definitions of positive allosteric modulation even in the absence of formal agonist concentration-response curve shift analysis (Christopoulos et al., 2014; Burford et al., 2015).

### β*-arrestin-2* recruitment

Compounds with measurable cAMP activity were further evaluated for β-arrestin-2 recruitment to assess signaling bias.

**FIGURE 4:**
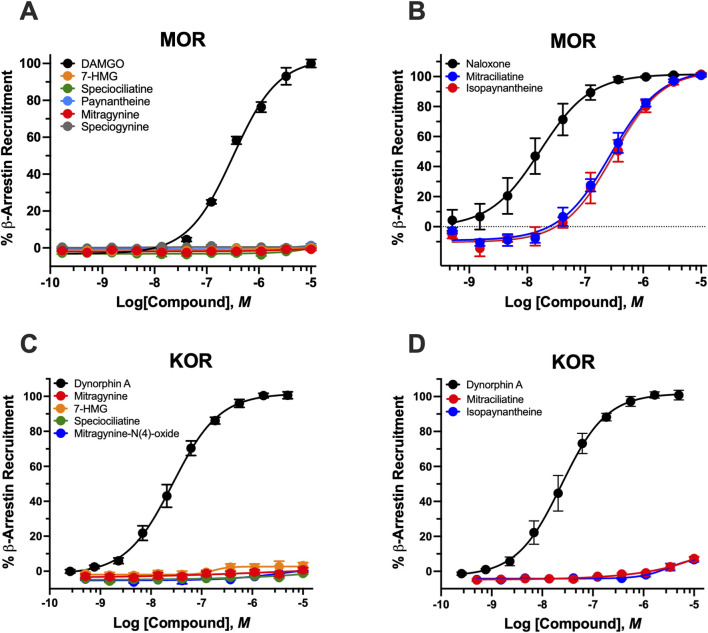
Recruitment of *β-arrestin-2* by kratom indole alkaloids at hMOR and hKOR*.* **(A)** Concentration-response curves showing the effects of the MOR full agonist DAMGO and select indole kratom alkaloids on *β-arrestin-2* recruitment in hMOR-expressing cells. Data are normalized to the maximal response produced by DAMGO and expressed as % E_max_. **(B)** Antagonist activity of naloxone and select indole kratom alkaloids evaluated by their ability to reverse met-enkephalin (E_80_ concentration) β-arrestin-2 recruitment. Responses are expressed as % naloxone E_max_. **(C,D)** Concentration-response curves showing the effects of the KOR full agonist Dynorphin A and select indole kratom alkaloids. Data are expressed as % E_max_. Data represent mean ± SEM.

*Indole Alkaloids* (Figure 4). Most indole alkaloids showed minimal or no β-arrestin-2 recruitment, consistent with G protein–biased signaling. At hMOR, only mitraciliatine and isopaynantheine exhibited measurable activity, functioning as antagonists with moderate potency (IC_50_ = of 281 ± 58 nM and 272 ± 43 nM, respectively) and complete inhibition of reference agonist–induced recruitment (Imax ∼100%). Neither compound showed measurable activity at hKOR.

7-HMG was the only indole alkaloid to induce β-arrestin-2 recruitment, displaying weak potency and partial efficacy selectively at hDOR (EC_50_ = 1644.5 ± 147.5 nM, E_max_ = 83.4 ± 0.4%) with no detectable recruitment at hMOR or hKOR. Notably, 7-HMG induced β-arrestin-2 recruitment only at hDOR despite showing cAMP agonist activity at all three receptors. All other tested alkaloids, including MG, speciogynine, speciociliatine, and their *N* (4)-oxides lacked measurable β-arrestin-2 activity across all receptor subtypes.

**FIGURE 5:**
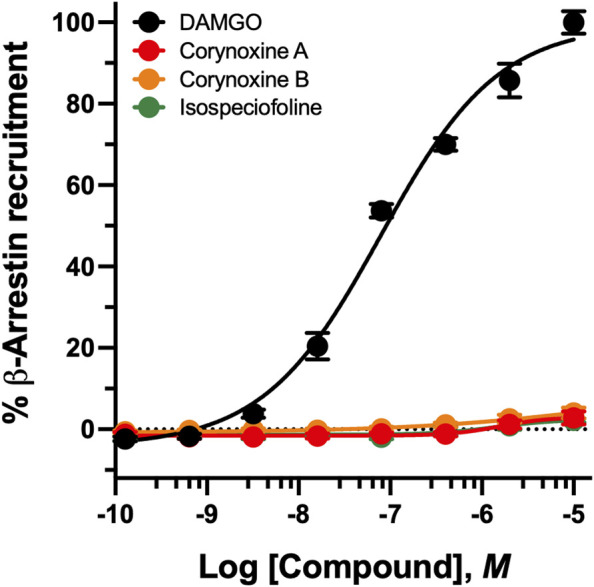
Recruitment of *β-arrestin2* by kratom oxindole alkaloids at hMOR*.* Concentration-response curves showing the effects of DAMGO and various oxindole kratom alkaloids on β-arrestin-2 recruitment in MOR-expressing cells. Data are normalized to the maximal response produced by DAMGO and expressed as % of E_max_. Data represent mean ± SEM.

*Oxindole alkaloids* (Figure 5). None of the oxindole alkaloids tested induced measurable β-arrestin-2 recruitment at hMOR, hKOR, or hDOR (EC_50_ > 10,000 nM. This included corynoxine A, corynoxine B, and isospeciofoline, which displayed potent hMOR agonism in cAMP assays. The complete absence of β-arrestin-2 recruitment despite robust G protein–mediated signaling indicates pronounced G protein bias across the oxindole class, distinguishing these compounds from classical opioid agonists.

GTPγS binding. Mitraciliatine and isopaynantheine displayed weak antagonist activity at the hMOR, characterized by low potency and partial efficacy. Mitraciliatine produced 74.6% *±* 3.7% maximal inhibition with an IC_50_ of 2818 ± 159 nM, while isopaynantheine produced 66.3% *±* 5.0% maximal inhibition with an IC_50_ of 4775 ± 1250 nM. In contrast, both alkaloids acted as moderate-potency, full-efficacy agonists at the hKOR. Mitraciliatine produced 84.8% ± 0.5% maximal stimulation with an EC_50_ of 654 ± 27 nM, while isopaynantheine produced 86.3% ± 3.4% stimulation with an EC_50_ of 536 ± 77 nM. These data indicate that both compounds act as weak μ-opioid antagonists but moderate κ-opioid agonists, revealing a clear functional divergence in their opioid receptor profiles. Quantitative [^35^S]GTPγS binding parameters for mitraciliatine and isopaynantheine are summarized in Supplementary Table S1.

### Structure-activity relationship (SAR) and docking analysis

**FIGURE 6:**
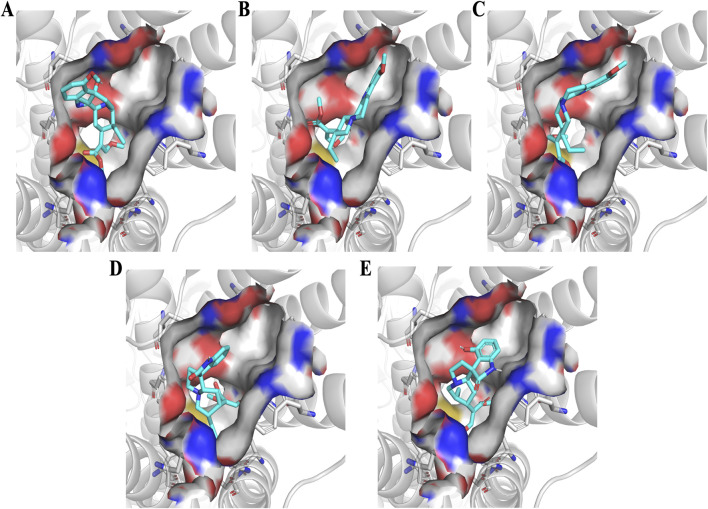
Docked orthosteric binding site occupation of hMOR by selected kratom alkaloids. Molecular docking was performed using AutoDock 4.2.6 with the active-state human MOR crystal structure (PDB: 7T2G). Representative binding poses showing the orthosteric pocket occupation by **(A)** 7-hydroxymitragynine, **(B)** speciogynine, **(C)** mitraciliatine, **(D)** corynoxine A, and **(E)** isospeciofoline. Protein is shown in cartoon representation with transmembrane helices colored by domain. Ligands are displayed as sticks with carbon atoms in cyan, nitrogen in blue, oxygen in red, and hydrogen in white. Key binding pocket residues are shown as lines. Poses represent the lowest-energy conformation from the most populated cluster (RMSD tolerance 2.0 Å).

The structure-activity relationships of the indole and oxindole alkaloids were further investigated through molecular docking using the agonist-bound structure of hMOR, crystallized with mitragynine pseudoindoxyl (PDB: 7T2G). Docked conformations of the compounds generally showed good agreement with reported crystal structures and ligand placements (Huang et al., 2015; Qu et al., 2023). The overall affinity trends across the indole and oxindole series were further characterized by the binding pocket’s accommodation of specific structural changes (Figure 6). The detailed SAR trends are provided in Supplementary Tables S2, S3.

**FIGURE 7:**
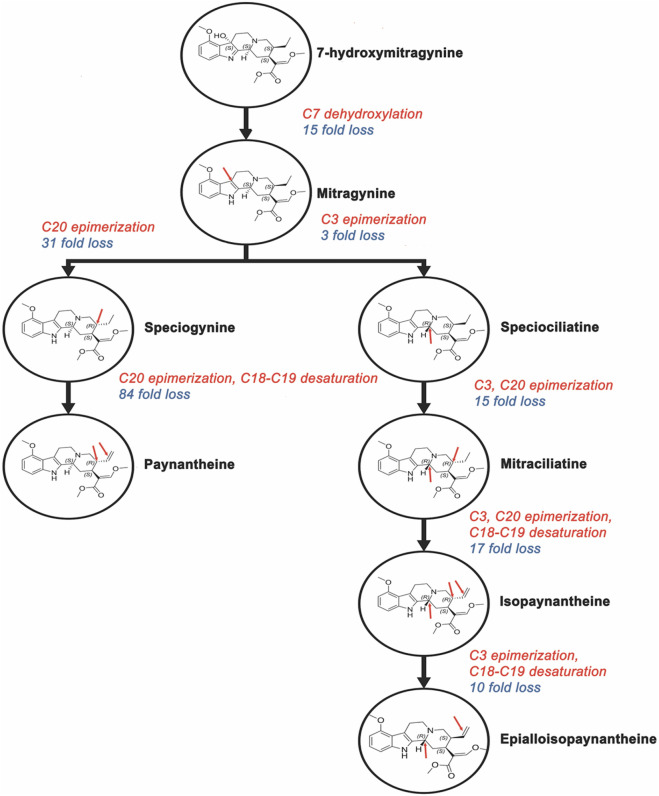
Indole kratom alkaloids affinity hierarchy chart. Structure-activity relationship analysis showing the impact of stereochemical modifications on hMOR binding affinity for indole alkaloids. Compounds are arranged in order of decreasing affinity (top to bottom) with fold changes calculated relative to 7-hydroxymitragynine (Ki = 15.1 nM, set as 1.0X). Red arrows indicate stereochemical inversions or structural modifications at key positions (C3, C7, C18-C19, C20) relative to the parent structure. The pharmacophoric elements conserved across high-affinity compounds include the C15 methyl acrylate and C20 ethyl substituent. Configuration shown in parentheses for modified stereocenters.

**FIGURE 8:**
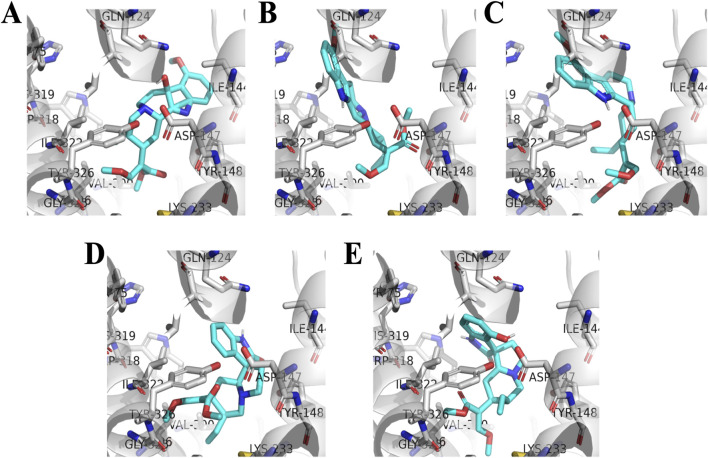
Residue interactions of hMOR with selected kratom alkaloids. Three-dimensional representations of docked ligands in the hMOR orthosteric binding pocket (PDB: 7T2G) showing key interacting residues for **(A)** 7-hydroxymitragynine, **(B)** speciogynine, **(C)** mitraciliatine, **(D)** corynoxine A, and **(E)** isospeciofoline. Ligands are shown as sticks with carbon atoms in cyan, nitrogen in blue, and oxygen in red. Protein residues are displayed in gray sticks with standard atom coloring (nitrogen in blue, oxygen in red, sulfur in yellow). Key binding site residues are labeled, including the conserved ASP147 (ionic interaction with protonated nitrogen), hydrophobic pocket residues (ILE144, LEU219, ILE322, VAL300, TYR326, LYS233), and polar residues (GLN124, TYR148). The protein backbone is shown as gray cartoon. Notable structural features include the 7-hydroxy group of 7-HMG (panel A) positioned for hydrogen bonding with ASN124, and conserved positioning of the C15 and C20 substituents within their respective hydrophobic sub-pockets across all compounds. Generated using PyMOL 2.5.

Indole Alkaloids. Docking of 7-HMG revealed several critical interactions with the hMOR binding pocket (Figures 7, 8). A direct hydrogen bond was observed between the 7-hydroxy group and ASN124 (3.1 Å). Other hydrogen bonds were noted with TYR148, TYR326, and LYS303, including a conserved interaction at ASP147. Significant hydrophobic associations occurred with LEU219, ILE296, ILE322, VAL300, MET151, TYR148, and TYR326. The side chain at position 20 was found to occupy a hydrophobic pocket formed by ILE296, ILE322, and TYR326. The methyl acrylate at position 15 occupied another pocket composed of MET141, HIS297, TYR148, TYR326, and TRP293, indicating a larger, potentially more flexible region. The overall conformation of 7-HMG, aside from the seven-position, largely retained similarity to that of mitragynine, suggesting that critical binding conformations are conserved. The approximately 15-fold reduction in hMOR affinity for MG (238 ± 28 nM) compared to 7-HMG is attributed to the loss of the hydrogen bond with ASN124.

**FIGURE 9:**
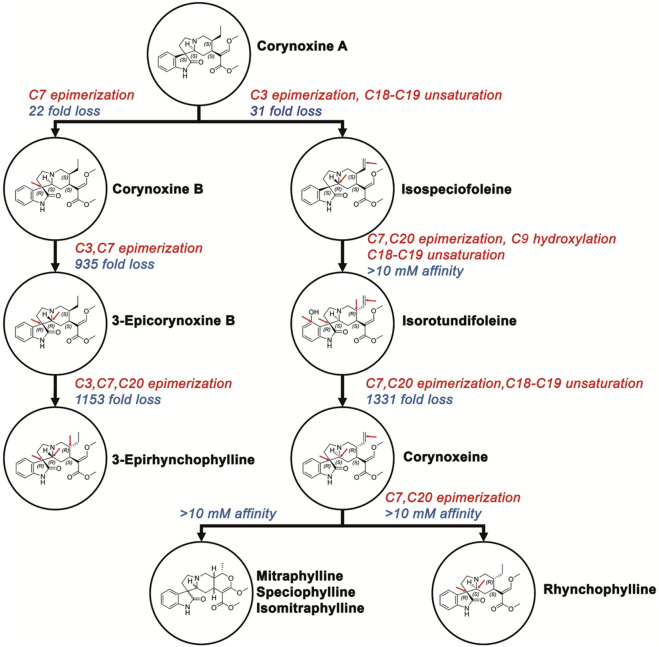
Oxindole kratom alkaloids affinity hierarchy chart. Hierarchical representation of binding affinity among oxindole alkaloids with corynoxine A (Ki = 5.4 nM) as the highest-affinity reference. Red arrows indicate stereochemical modifications with fold changes in affinity annotated along each branch. Single stereochemical inversions produce 22- to 31-fold affinity losses, while multiple inversions result in 935- to 1331-fold decreases or loss of measurable binding (Ki > 10 μM, bottom tier). Despite greater conformational flexibility compared to indole alkaloids, conserved C15 and C20 pharmacophoric elements remain critical determinants of hMOR affinity. Chemical structures shown within circles display key stereochemical configurations.

Inversion of the hydrogen at position 20 in MG (i.e., from *S* to *R*), forming speciogynine, resulted in an approximately 2-fold decrease in affinity. The major difference in the docked poses of these two compounds stemmed from the indole moiety’s occupation of the pocket formed by TM1, 2, and 7 (Figure 9), and the altered orientation of the methyl acrylate at C15. Further conformational changes impacting the C20 ethyl group were observed. While the transition from C20 ethyl to ethene (*i.e.*, from mitraciliatine to isopaynantheine) showed a negligible effect on affinity, the inversion of C20 back to the optimal pro-*S* configuration, as seen in epiallo-isopaynantheine, yielded another high-affinity indole. Changes in the configuration at C3 (3*R*) significantly affected the fit of compounds such as speciogynine in the pocket, creating a large gap between the ligand and TM7.

**FIGURE 10:**
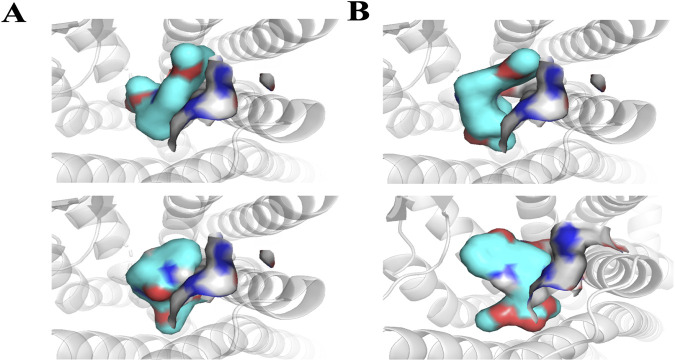
Ligand interactions with transmembrane helix 7 (TM7) of hMOR. **(A)** Top: speciogynine; bottom: corynoxine A, both docked into active hMOR (PDB: 7T2G), demonstrating how indole versus oxindole scaffolds occupy the TM7 interface region differently. **(B)** Top: mitraciliatine docked into active hMOR (7T2G) showing agonist-compatible binding orientation; bottom: mitraciliatine docked into inactive hMOR (PDB: 4DKL) showing antagonist-compatible conformation. Ligands are displayed as molecular surfaces with cyan carbon, blue nitrogen, and red oxygen. Protein shown as a gray cartoon with the binding pocket surface in transparent gray. The altered positioning against TM7, particularly influenced by stereochemistry at C3, correlates with changes in binding affinity and functional profile. Generated using PyMOL 2.5.

**FIGURE 11:**
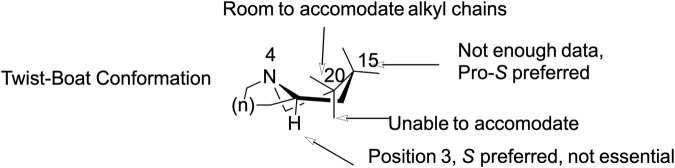
General pharmacophore model for hMOR-binding kratom alkaloids. Key structural determinants of hMOR binding derived from SAR and docking analysis: basic nitrogen at position four (ionic interaction with ASP147), positions 15 and 20 accommodate essential alkyl chains in hydrophobic sub-pockets (C15 methyl acrylate, C20 ethyl/ethene preferred, pro-S configuration optimal), and position three shows stereochemical preference (S preferred but not essential). This pharmacophore framework applies across both indole and oxindole alkaloid series.

Oxindole Alkaloids. The oxindole scaffold, possessing increased degrees of freedom, exhibited more pronounced conformational changes upon stereochemical inversion, which correlated with larger swings in affinity (Figure 10). A notable trend was the almost total loss of affinity when two or more chiral centers were in the R configuration. Corynoxine A, the highest affinity oxindole alkaloid, showed significant overlap with 7-HMG at the C15 and C20 positions in its docked conformation (Figure 7). It maintained interactions with residues such as LEU219, ILE296, ILE322, VAL300, MET151, TYR148, TYR326, MET141, HIS297, TYR148, and TRP293. Conversely, the oxindole moieties accessed different pockets and displayed greater variability in their interactions. For example, the oxindole corynoxine A interacted with ASN124, TYR326, ILE144, and TYR148, while isospeciofoline interacted with ASN124, TRP318, TYR326, ILE322, and was positioned for hydrogen bonding with ASP147. Despite differences in the oxindole core, the affinity data for positions 15 and 20 followed a similar trend between indoles and oxindoles, allowing the postulation of a general SAR and pharmacophore (Figure 11).

## Discussion

The present study provides the most comprehensive evaluation to date of *M. speciosa* indole and oxindole alkaloids at hMOR, hKOR and hDOR, integrating binding, functional, and computational analyses to clarify their pharmacological diversity. While previous investigations have established that major alkaloids like MG and 7-HMG act as G-protein-biased partial agonists at hMOR (Kruegel et al., 2016; Varadi et al., 2016; Ellis et al., 2020; Obeng et al., 2020) these studies typically focused on a limited number of compounds and often employed rodent receptor models. By extending this framework to a diverse set of minor alkaloids under uniform experimental conditions using human receptors, we reveal previously unrecognized patterns of efficacy, selectivity, and signaling bias.

Our findings confirm and extend the G-protein-biased agonist profiles of MG and 7-HMG while revealing novel pharmacological diversity across the alkaloid class. Several indole alkaloids - including speciociliatine, paynantheine, and epiallo-isopaynantheine - exhibited measurable hMOR and/or hKOR activity, whereas oxindole congeners displayed predominantly hMOR-selective profiles. Our characterization of speciociliatine as a partial agonist at hMOR (E_max_ ∼73%) reconciles prior conflicting reports that describe it as either a full agonist (Chear et al., 2021) or a weak antagonist (Kruegel et al., 2016; Zhou et al., 2021). These discrepancies likely reflect differences in assay systems, receptor expression levels, and reference compound normalization, underscoring the importance of standardized methodology across human receptor models. Beyond these orthosteric ligands, we identified speciophylline as a positive allosteric modulator at hMOR that lacks detectable orthosteric binding - distinguishing it from alkaloids like MG and 7-HMG, which exhibit primarily orthosteric activity with possible secondary allosteric effects (Zhou et al., 2021). We also characterized mitraciliatine and isopaynantheine as mixed MOR antagonists/KOR agonists at human receptors, refining earlier reports of MOR partial agonism observed in mouse receptor assays (Chakraborty et al., 2021). These receptor-level distinctions provide a mechanistic framework for interpreting kratom’s complex and variable pharmacological effects.

### Functional diversity and signaling bias

The predominant functional profile observed across kratom alkaloids—partial agonism at hMOR with minimal β-arrestin-2 recruitment - extends previous findings for MG and 7-HMG (Varadi et al., 2016; Kruegel and Grundmann, 2018) to a structurally diverse set of indole and oxindole alkaloids. This G-protein signaling bias, which we confirmed across multiple assay platforms (cAMP inhibition, β-arrestin-2 recruitment, [^35^S]GTPγS binding), distinguishes kratom alkaloids from classical opioids like morphine and fentanyl that strongly recruit β-arrestin-2. While earlier studies suggested that β-arrestin-2 recruitment mediates respiratory depression (Raehal et al., 2005), this hypothesis has been challenged by recent work demonstrating comparable respiratory depression in β-arrestin-2 knockout mice (Kliewer et al., 2020; Bachmutsky et al., 2021). Nevertheless, G-protein-biased agonists may still offer therapeutic advantages through other mechanisms, such as reduced receptor desensitization and tolerance (Schmid et al., 2017). This widespread signaling bias is consistent with kratom’s reportedly lower incidence of respiratory depression compared to traditional opioids, though direct causal links remain to be established *in vivo*.

Beyond confirming biased agonism, our functional characterization revealed unexpected pharmacological diversity. Mitraciliatine and isopaynantheine exhibited mixed profiles - antagonism at hMOR coupled with moderate agonism at hKOR. This profile may provide analgesia while avoiding both the euphoria associated with MOR agonism and the dysphoria produced by full KOR activation, challenges that have driven interest in biased and mixed opioid ligands (Brust et al., 2016; Schmid et al., 2017). Most oxindole alkaloids, particularly corynoxine A, corynoxine B, and isospeciofoline, displayed potent hMOR agonism (EC_50_ values 38–276 nM) with complete absence of β-arrestin-2 recruitment, representing an extreme form of G-protein bias.

An additional consideration relates to receptor trafficking and internalization. Although several kratom alkaloids exhibited minimal or no β-arrestin-2 recruitment, hMOR internalization was not directly assessed in this study. Importantly, reduced β-arrestin recruitment does not necessarily preclude receptor internalization, as arrestin-independent internalization mechanisms have been described for MOR (Whistler et al., 1999; Williams et al., 2013). Consequently, the possibility remains that some kratom alkaloids promote receptor internalization through alternative pathways. Future studies directly assessing receptor trafficking and internalization will be necessary to determine how these signaling profiles translate to receptor regulation and downstream cellular responses.

Speciophylline’s unique positive allosteric modulation (Figures 3A–C; Table 2) offers a mechanistically distinct approach to opioid receptor modulation. By enhancing endogenous opioid signaling without directly activating the receptor, speciophylline contrasts with the orthosteric agonist and antagonist profiles observed for other indole and oxindole alkaloids in this study. While the structural basis for this activity remains undefined, speciophylline’s oxindole scaffold and lack of measurable orthosteric binding distinguish it from other kratom alkaloids and suggest interaction with a site outside the canonical orthosteric pocket. The specific allosteric binding site engaged by speciophylline remains to be determined, and whether it overlaps with sites described for established μ-opioid receptor PAMs will require dedicated mechanistic studies, including mutagenesis, structural approaches, and PAM co-application analyses. This profile aligns with emerging interest in allosteric modulation as a strategy to fine-tune opioid receptor signaling while preserving spatial and temporal control by endogenous ligands. Collectively, these findings underscore that kratom’s complex pharmacology arises from the combined actions of alkaloids with divergent receptor selectivities, efficacies, and signaling biases rather than from a single dominant mechanism.

### Structure–activity relationships

To understand the structural basis for this pharmacological diversity, the structure-activity relationships (SAR) observed across the indole and oxindole series are supported by molecular docking in the agonist-bound hMOR crystal structure (PDB: 7T2G), which has been widely used to examine ligand-specific receptor conformations and signaling outcomes (Huang et al., 2015; Qu et al., 2023). Docked poses for the compounds in this study recapitulated key interactions within the orthosteric pocket, including engagement of ASP147 and surrounding hydrophobic residues, consistent with prior computational studies of kratom alkaloids (Kruegel et al., 2016; Ellis et al., 2020; Zhou et al., 2021). A central SAR finding is the conserved role of substituents at C15 and C20 across both indole and oxindole scaffolds: the C15 methyl acrylate and C20 ethyl/ethene side chains consistently occupy conserved hydrophobic sub-pockets. The overlap of docked conformations for 7-HMG and corynoxine A suggests C15 and C20 are critical anchors for productive hMOR binding, consistent with earlier reports that mitragynine-derived ligands exploit these regions to stabilize distinct active states (Varadi et al., 2016; Zhou et al., 2021; Qu et al., 2023).

The pronounced impact of seven-position hydroxylation and stereochemistry on affinity and efficacy is well rationalized by the docking models. Hydroxylation of MG to form 7-HMG markedly increased hMOR affinity, which our models attribute to the formation of a direct hydrogen bond with ASN124 (3.1 Å), consistent with previous proposals that polar contacts at this residue enhance agonist potency (Huang et al., 2015; Kruegel et al., 2016; Zhou et al., 2021). The approximate 15-fold affinity improvement observed for 7-HMG relative to MG directly reflects that additional interaction, while other key binding determinants remain conserved. Stereochemical effects at C3 and C20 were particularly informative. Inversion at C20 (e.g., MG vs. speciogynine) altered how the indole ring system occupied the TM1-TM2-TM7 region and reoriented the C15 methyl acrylate, producing marked reductions in affinity that mirror previous SAR reports for mitragynine analogs (Kruegel et al., 2016; Varadi et al., 2016). Conversely, epimerization at C3, which distinguishes speciociliatine from MG, was associated with improved hMOR binding in our assays and a more favorable fit in the docking models, consistent with prior observations that C3 configuration is a key determinant of MOR activity (Varadi et al., 2016; Obeng et al., 2020; Chear et al., 2021; Zhou et al., 2021).

The oxindole scaffold, with greater conformational flexibility, exhibited more pronounced structural changes upon stereochemical inversion, correlating with greater variations in affinity. A notable trend was the almost complete loss of affinity when two or more chiral centers adopted the R configuration, often disrupting key contacts with ASN124, TYR326, and ILE322. Corynoxine A, the highest affinity oxindole moiety, accessed distinct oxindole sub-pockets with more variable polar contacts than the indole scaffold. The strong affinity of corynoxine A underscores how shared peripheral pharmacophoric elements (C15 and C20) can be combined with scaffold-specific interactions to tune receptor engagement. Together with existing docking and pharmacological studies, these findings support a general pharmacophore in which a conserved basic center, C15/C20 hydrophobic substituents, and configuration-dependent orientation of the indole/oxindole core jointly dictate MOR affinity, subtype selectivity, and potentially signaling bias (Figure 11).

While docking analyses provide insight into binding determinants, they do not fully predict functional outcomes. Compounds with similar binding affinities exhibited divergent efficacies, ranging from high efficacy agonism (corynoxine A) to antagonism (mitraciliatine, isopaynantheine) at hMOR. These differences likely reflect ligand-specific stabilization of distinct receptor conformational states that cannot be captured by static docking models. For example, the antagonist activity of mitraciliatine and isopaynantheine, despite moderate binding affinity, suggests these compounds preferentially stabilize inactive receptor conformations, consistent with their docked poses in the inactive hMOR structure (4DKL). Future molecular dynamics simulations may clarify how subtle structural variations translate to divergent signaling outcomes.

### Translational implications

The widespread G-protein bias observed across kratom alkaloids represents a notable and consistent pharmacological feature when compared with classical opioids. Reports of reduced respiratory depression and lower abuse liability associated with kratom use (Hemby et al., 2018; Yue et al., 2018; Chakraborty et al., 2021; Henningfield et al., 2022; Hill et al., 2022) have prompted interest in whether such outcomes might relate to biased signaling profiles; however, current evidence does not support a direct or uniform causal relationship between reduced β-arrestin-2 recruitment and improved safety. Our findings demonstrate that G-protein bias with minimal β-arrestin-2 recruitment extends beyond mitragynine and 7-hydroxymitragynine to include structurally diverse indole and oxindole alkaloids, including potent oxindole agonists that lack detectable β-arrestin-2 recruitment. While these shared signaling features warrant further investigation, the extent to which *in vitro* signaling bias translates to *in vivo* safety or therapeutic advantage remains incompletely understood. Additional preclinical studies will be required to determine whether individual alkaloids, such as corynoxine A, exhibit favorable therapeutic windows in animal models.

Beyond MOR-biased agonism, our identification of pharmacologically distinct kratom alkaloids highlights opportunities for developing novel analgesic strategies. Several compounds merit further optimization as potential non-addictive analgesics: mitraciliatine and isopaynantheine as mixed MOR antagonist/KOR agonist templates, and hKOR-selective N-oxide derivatives as partial agonists that may retain antinociceptive efficacy while mitigating the dysphoria and sedation associated with full KOR activation (Carlezon et al., 2006; Knoll and Carlezon, 2010; Brust et al., 2016). Most notably, speciophylline’s pure positive allosteric modulation of hMOR represents a mechanistically distinct profile among kratom alkaloids and aligns with emerging interest in allosteric modulators as a strategy to enhance opioid analgesia while limiting tolerance and dependence (Burford et al., 2013; Burford et al., 2015; Livingston et al., 2018).

The structure-activity relationships elucidated here provide a rational framework for medicinal chemistry optimization. Stereochemical modifications at key positions (e.g., C3, C20) can switch compounds between agonist, antagonist, and receptor-selective profiles, enabling targeted development of G-protein-biased ligands with minimized β-arrestin-2 recruitment. These naturally occurring scaffolds thus offer structurally tunable templates for designing safer opioid therapeutics, though their ultimate clinical utility will depend on *in vivo* validation of efficacy and safety profiles.

### Limitations and future directions

Several key limitations must be acknowledged. First, our *in vitro* assays, while enabling systematic comparison across human receptor subtypes, do not capture the physiological complexity of *in vivo* systems, including receptor distribution, neuronal circuitry, adaptive signaling, or the impact of receptor reserve on functional outcomes. Second, metabolic considerations remain incompletely addressed. The CYP3A4-mediated conversion of MG to 7-HMG in humans (Kruegel et al., 2019; Obeng et al., 2020) and potential contributions of other metabolites were not directly examined here, and species differences in bioactivation complicate cross-species extrapolation (Kruegel and Grundmann, 2018). Third, kratom alkaloids exhibit poly-pharmacology beyond opioid receptors, including interactions with adrenergic, serotonergic, and purinergic systems (Kruegel et al., 2016; Obeng et al., 2020; Chear et al., 2021; Leon et al., 2021), which likely contribute to kratom’s stimulant and mood-altering effects but were outside the scope of this investigation. Finally, our findings characterize individual purified alkaloids, whereas kratom users consume complex mixtures with variable alkaloid ratios; potential synergistic or antagonistic interactions among alkaloids remain unexplored.

Validating these receptor-level findings in *in vivo* preclinical models is critical. Studies using humanized opioid receptor knock-in mice could clarify whether G-protein bias translates to improved safety profiles *in vivo. At* the same time, behavioral pharmacology experiments can assess analgesic efficacy, abuse liability, and withdrawal severity for promising candidates like corynoxine A and speciophylline. Structural biology approaches, such as co-crystallization of alkaloid-receptor complexes and molecular dynamics simulations, would provide atomic-level insights into how stereochemistry determines signaling bias and could guide rational design of optimized analogs. Additionally, investigating alkaloid combinations at physiologically relevant ratios may reveal synergistic interactions that contribute to whole-plant kratom effects. Ultimately, integrating receptor pharmacology with pharmacokinetics, metabolism, and *in vivo* efficacy/toxicity studies will be essential for defining the therapeutic potential and safety boundaries of *M. speciosa* alkaloids.

An additional consideration is the emerging evidence that MOR signaling can involve differential coupling to distinct Gα subtypes, including Gαz, which may contribute to signaling outcomes not fully captured by canonical Gi/o-based assays (Masuho et al., 2015; Gillis et al., 2020). The functional assays used in this study (cAMP inhibition, β-arrestin-2 recruitment, and [^35^S]GTPγS binding) report aggregate G-protein signaling and do not resolve Gα subtype-specific coupling. As a result, potential contributions of Gαz-mediated signaling to the pharmacological profiles of kratom alkaloids were not directly assessed. Future studies employing G-protein subtype resolved platforms, such as TRUPATH, will be critical for further delineating the signaling mechanisms underlying the observed bias profiles.

## Conclusion

This systematic characterization of kratom alkaloids at human opioid receptors reveals a pharmacologically diverse class of natural products engaging multiple mechanisms, such as partial agonism, G-protein-biased signaling, mixed agonist-antagonist activity, and allosteric modulation. The widespread G-protein bias with minimal β-arrestin-2 recruitment offers a plausible mechanistic explanation for kratom’s atypical safety profile relative to classical opioids (Hemby et al., 2018; Kruegel and Grundmann, 2018; Obeng et al., 2020). Beyond this shared property, the identification of receptor-selective alkaloids and speciophylline’s distinct positive allosteric modulation highlights structurally diverse opportunities for developing safer analgesics. These scaffolds show potential for developing analgesics with reduced respiratory depression, though effects on dependence liability require *in vivo* validation. Our findings establish structure-function relationships that link alkaloid stereochemistry to receptor pharmacology, signaling bias, and functional outcomes. This mechanistic framework provides a foundation for both evidence-based regulatory assessment and rational development of kratom-derived therapeutics.

## Data Availability

The raw data supporting the conclusions of this article will be made available by the authors, without undue reservation.
