# Supplementary material for: Multifaceted modulation of human opioid receptors by kratom alkaloids: binding affinity, functional selectivity, and allosteric activity
Source: Front Pharmacol. 2026 Mar 17;17:1763551. doi: 10.3389/fphar.2026.1763551 (PMC13036161; doi:10.3389/fphar.2026.1763551)
Supplement: Supplementary file 1 [file Table1.pdf]

Supplementary Table 1: [^{35}S]GTPγS binding activity of mitraciliatine and isopaynantheine at hMOR and hKOR. [^{35}S]GTPγS binding assays were used to assess G-protein activation by mitraciliatine and isopaynantheine at hMOR and hKOR. At hMOR, both compounds were evaluated in antagonist mode and inhibited DAMGO-stimulated G-protein activation; IC_{50} values and maximal inhibition (I_{max}) are shown. At hKOR, compounds were evaluated in agonist mode, stimulating G-protein activation; EC_{50} values and maximal stimulation (E_{max}) are shown, normalized to the response produced by the reference full agonist U-69,593. Data are presented as mean ± SEM from ≥3 independent experiments.

	**hMOR**	**hKOR**
	**IC50, nM (*Imax*,%)**	**EC50, nM (*Emax*,%)**
Mitraciliatine	2818 ± 159 (*74.6 ± 3.7)*	654 ± 27 (*84.8 ± 0.5)*
Isopaynantheine	4775 ± 1250 (*66.3 ± 5.0)*	536 ± 77 (*86.3 ± 3.4)*
