# Supplementary material for: Multifaceted modulation of human opioid receptors by kratom alkaloids: binding affinity, functional selectivity, and allosteric activity
Source: Front Pharmacol. 2026 Mar 17;17:1763551. doi: 10.3389/fphar.2026.1763551 (PMC13036161; doi:10.3389/fphar.2026.1763551)
Supplement: Supplementary file 2 [file Table2.pdf]

Supplementary Table 2. Essential stereochemistry of binding affinities for kratom indole alkaloids. Structure-activity relationship analysis showing the impact of stereochemical configuration at key positions (C3, C7, C15, C20) on hMOR binding affinity for indole alkaloids. Compounds are organized by structural similarity with stereochemical configurations indicated. Ki values (nM) determined by competitive radioligand binding against [^{3}H]-DAMGO in CHO-K1 cells expressing hMOR. Fold changes calculated relative to 7-hydroxymitragynine (Ki = 15.1 nM). Stereochemical inversions at C20 and modifications at C7 produce the most significant affinity changes, while C15 methyl acrylate remains conserved across all high-affinity compounds. ND = not detected (Ki >10 μM). Data represent mean ± SEM from n≥3 independent experiments.

Indole Alkaloid	7	9	3	15	19	20	MOR Affinity	KOR Affinity	DOR Affinity
7-hydroxymitragynine	OH	OCH3	*S*	*S*	CH3	*S*	15.1 ± 3.7 nM	597 ± 37 nM	137.3 ± 21.3 nM
Mitragynine	-	OCH3	*S*	*S*	CH3	*S*	238 ± 28 nM	482 ± 29 nM	>10 μM
Speciociliatine	-	OCH3	*R*	*S*	CH3	*S*	49 ± 13 nM	312 ± 56 nM	>10 μM
Speciogynine	-	OCH3	*S*	*S*	CH3	*R*	472 ± 65 nM	>10 μM	3355 ± 102 nM
Paynantheine	-	OCH3	*S*	*S*	CH2	*R*	1270 ± 141 nM	>10 μM	9029 ± 2436 nM
Mitraciliatine*	-	OCH3	*R*	*S*	CH3	*R*	226 ± 26 nM	108 ± 16 nM	>10 μM
Isopaynantheine*	-	OCH3	*R*	*S*	CH2	*R*	262 ± 36 nM	130 ± 12 nM	2876 ± 848 nM
Epiallo-isopaynantheine*	-	OCH3	*R*	*S*	CH2	*S*	155 ± 15 nM	120 ± 8 nM	>10 μM

**antagonist*
