# Supplementary material for: Multifaceted modulation of human opioid receptors by kratom alkaloids: binding affinity, functional selectivity, and allosteric activity
Source: Front Pharmacol. 2026 Mar 17;17:1763551. doi: 10.3389/fphar.2026.1763551 (PMC13036161; doi:10.3389/fphar.2026.1763551)
Supplement: Supplementary file 3 [file Table3.pdf]

Supplementary Table 3. Essential stereochemistry of binding affinities for kratom oxindole alkaloids. Structure-activity relationship analysis showing the impact of stereochemical configuration at key positions (C3, C7, C15, C20, C22) on hMOR binding affinity for oxindole alkaloids. Compounds are organized by structural similarity, with stereochemical configurations indicated. Fold changes calculated relative to corynoxine A (Ki = 5.4 nM). The oxindole scaffold exhibits greater sensitivity to stereochemical changes than indoles, with compounds possessing two or more non-optimal configurations showing a loss of measurable binding (Ki > 10 μM). Despite conformational flexibility of the oxindole core, conserved C15 and C20 elements remain critical for receptor engagement. Data represent mean ± SEM from n≥3 independent experiments. Dashes (--) denote no data available.

Oxindole Alkaloid	7	9	3	15	19	20	MOR Affinity	KOR Affinity	DOR Affinity
Corynoxine A	*S*	H	*S*	*S*	CH3	*S*	5.4 ± 0.3 nM	2310 ± 325 nM	2932 ± 1110 nM
Corynoxine B	*R*	H	*S*	*S*	CH3	*S*	118.3 ± 12.1 nM	--	--
Isospeciofoleine	*S*	OH	*R*	*S*	CH3	*S*	167 ± 13.3 nM	>10 μM	8050 ± 1340 nM
Isorotundifoeline	*R*	OH	*S*	*S*	CH2	*R*	>10 μM	>10 μM	>10 μM
3-Epicorynoxine B	*R*	H	*R*	*S*	CH3	*S*	5050 ± 1403 nM	>10 μM	6665 ± 3022 nM
3-Epirhyncophylline	*R*	H	*R*	*S*	CH3	*R*	6226 ± 771 nM	>10 μM	>10 μM
Corynoxeine	*R*	H	*S*	*S*	CH2	*R*	7187 ± 865 nM	>10 μM	>10 μM
Rhynchophylline	*R*	H	*S*	*S*	CH3	*R*	>10 μM	>10 μM	4246 ± 551 nM
Mitraphylline	*R*	H	*S*	*S*	*Constrained*	*Constrained*	>10 μM	>10 μM	>10 μM
Speciophylline	*S*	H	*R*	*R*	*Constrained*	*Constrained*	>10 μM	>10 μM	>10 μM
Isomitraphylline	*S*	H	*R*	*S*	*Constrained*	*Constrained*	>10 μM	>10 μM	>10 μM
